# Patch Testing Under Biologics: Six Cases From a Tertiary Centre

**DOI:** 10.1111/cod.70065

**Published:** 2025-12-14

**Authors:** Alessandra Chiei‐Gallo, Francesca Barei, Angelo V. Marzano, Silvia M. Ferrucci

**Affiliations:** ^1^ Department of Pathophysiology and Transplantation Università Degli Studi Di Milano Milan Italy; ^2^ Dermatology Unit Fondazione IRCCS Ca' Granda Ospedale Maggiore Policlinico Milan Italy

Allergic contact dermatitis (ACD) is a T cell–mediated (type IV delayed‐type) hypersensitivity reaction induced by the immunogenic properties of a subset of chemicals and requires the activation of both innate and acquired immunity. Biologic therapies targeting cytokines such as tumour necrosis factor (TNF)‐α, IL‐12/23 and IL‐17 are widely used in treating various chronic inflammatory conditions. Their immunomodulatory effects have raised concerns about potential suppression of patch test (PT) responses, possibly leading to false‐negative results [1, 2, 3]. Nonetheless, clinical evidence, including case series and expert opinions, suggests that positive PT reactions can still occur during biologic treatment [1, 4, 5, 6]. Patch testing during treatment with anti‐TNFα agents and guselkumab has been reported; however, to our knowledge, no studies have been published on PT outcomes in patients receiving bimekizumab or risankizumab.

## 1 Methods/Materials

We herein present our experience with patch testing in six patients affected by psoriasis, hidradenitis suppurativa (HS) or Crohn's disease, treated with bimekizumab (*n* = 1) for psoriasis and HS, risankizumab (*n* = 1) for psoriasis, guselkumab (*n* = 2) for psoriasis and Adalimumab (*n* = 2) for Crohn's disease. These patients were referred to our tertiary centre for suspected ACD. Patch testing was performed without discontinuing biologic therapy, using the T.R.U.E. TEST system (Smart Practice, Phoenix, AZ, USA), which includes Panels 1.3, 2.3 and 3.3, comprising 35 common allergens and a negative control.

## 2 Results

The clinical and epidemiological characteristics are detailed in Table 1. Five of six patients demonstrated at least one positive reaction, most commonly to nickel sulphate, with additional sensitisations to cobalt, chromium, and gold sodium thiosulfate. All reactions were clinically relevant.

## 3 Discussion

These findings align with previous studies reporting preserved PT reactivity despite systemic immunomodulation [1, 2, 3]. Although biologics target Th1/Th17 pathways involved in ACD pathogenesis, our results support previous reports showing that patch test reactivity may persist during biologic treatment. While positive reactions to metals were detected, weaker or doubtful responses (particularly to allergens such as fragrances or preservatives) could have been suppressed by biologic therapy. This suggests that negative results should be interpreted with caution. Our experience supports the feasibility and clinical usefulness of performing patch testing during biologic therapy, although it remains uncertain whether biologics may attenuate certain elicitation reactions or reduce the intensity of weak responses. The use of the TRUE Test panel alone (without additional series) and the absence of a control group limit our ability to fully assess test sensitivity and generalise the findings. This highlights the need for future prospective studies, ideally including patch testing both before starting biologic therapy and after several months of treatment. Nevertheless, given the small and heterogeneous sample, these findings should be viewed as preliminary, and further controlled studies are needed to better define how biologics might influence patch test reactivity.

**TABLE 1:** Clinical and epidemiological characteristics of the population.

Patients	Biologic	Disease	Sex, age	Work	Hobbies	Other comorbidities	Dermatitis localisation	Dermatitis duration	Series	Results	Relevance
Patient 1	Bimekizumab^a^	HS and Pso	F, 37	Office work	Not relevant	None	Lips	5 years	True test series	Nickel Sulphate (3+) Cobalt chloride (2+)	Past relevance
Patient 2	Risankizumab^b^	Pso	M, 49	Metalworking	Not relevant	Dyslipidaemia, anxiety	Feet	8 months	True test series	Nickel Sulphate (1+)	Past relevance
Patient 3	Guselkumab^c^	Pso	F, 56	Office work	Not relevant	Fibromyalgia	Hands and feet	3 years	True test series	Nickel Sulphate (2+)	Relevant for sulphate nickel
Patient 4	Guselkumab^c^	Pso	F, 63	Office work	Not relevant	Hypertension, dyslipidaemia, hepatic steatosis	Hands and feet	8 years	True test series	Nickel Sulphate (1+) Potassium dichromate (2+) Cobalt chloride (2+)	Relevant for sulphate nickel and potassium dichromate
Patient 5	Adalimumab^d^	Crohn's disease and spondylarthritis	M, 74	Typesetter	Not relevant	Vitiligo, BPH, CSU	Abdomen, superior limbs, inferior limbs	10 months	True test series	Nickel Sulphate (3+) Gold sodium thiosulfate (1+)	Relevant
Patient 6	Adalimumab^d^	Crohn's disease	F, 21	Teacher	Not relevant	Erythema nodosum and uveitis in 2023; allergic conjunctivitis	Lips	2 months	True test series	Negative	—

*Note*: Bimekizumab posology^a^: 320 mg at Weeks 0, 4, 8, 12, and 16, then every 8 weeks thereafter; Risankizumab posology^b^: 160 mg at Week 0, followed by 80 mg every 4 weeks; Guselkumab posology^c^: 100 mg at Weeks 0 and 4, then every 8 weeks thereafter; Adalimumab posology^d^: 80 mg at Week 0, then 40 mg every 2 weeks. Patch tests were applied under 48‐hour occlusion, and the results reported in the table refer to the Day‐4 (96‐hour) reading. Allergen specifications (concentrations and vehicles): nickel sulphate (200 µg/cm², hydroxypropyl cellulose), potassium bichromate (54 µg/cm², povidone), cobalt chloride (20 µg/cm², hydroxypropyl cellulose), and gold–sodium thiosulfate (75 µg/cm², hydroxypropyl cellulose).

Abbreviations: BPH, benign prostatic hyperplasia; CSU, chronic spontaneous urticaria; F, female; HS, hidradenitis suppurativa; M, male; PsA, psoriatic arthritis; Pso, psoriasis.

## Author Contributions

**Francesca Barei:** writing – review and editing. **Alessandra Chiei‐Gallo:** conceptualization, investigation, writing – review and editing, writing – original draft. **Angelo V. Marzano:** writing – review and editing, writing – original draft.

## Funding

This work was supported by Fondazione IRCCS Ca' Granda Ospedale Maggiore Policlinico and Ministero della Salute.

## Ethics Statement

This is a retrospective study on a small population; no ethical approval is needed. The patients in this manuscript have given written informed consent to the publication of their case details.

## Conflicts of Interest

Silvia M. Ferrucci is the principal investigator in a clinical trial to Amgen, Sanofi, Novartis, Lilly, Leo Pharma, Abbvie and she is on the advisory board or a speaker for Novartis, Menarini, Sanofi, Abbvie and Leo Pharma. Francesca Barei has a conflicts of interest with Leo Pharma and Almirall. The other authors declare that there is no conflicts of interest.

## Data Availability

Data sharing not applicable to this article as no datasets were generated or analysed during the current study.
